# Upregulated SLC22A3 has a potential for improving survival of patients with head and neck squamous cell carcinoma receiving cisplatin treatment

**DOI:** 10.18632/oncotarget.20637

**Published:** 2017-09-04

**Authors:** Cheng-Ming Hsu, Pai-Mei Lin, Jan-Gowth Chang, Hsin-Ching Lin, Shau-Hsuan Li, Sheng-Fung Lin, Ming-Yu Yang

**Affiliations:** ^1^ Department of Otolaryngology, Chiayi Chang Gung Memorial Hospital and Chang Gung University College of Medicine, Chiayi, Taiwan; ^2^ Department of Otolaryngology, Kaohsiung Chang Gung Memorial Hospital and Chang Gung University College of Medicine, Kaohsiung, Taiwan; ^3^ Graduate Institute of Clinical Medical Sciences, College of Medicine, Chang Gung University, Tao-Yuan, Taiwan; ^4^ Department of Nursing, I-Shou University, Kaohsiung, Taiwan; ^5^ Department of Laboratory Medicine, China Medical University Hospital, Taichung, Taiwan; ^6^ College of Medicine, China Medical University, Taichung, Taiwan; ^7^ Epigenome Research Center, China Medical University Hospital, Taichung, Taiwan; ^8^ Division of Hematology-Oncology, Department of Internal Medicine, Kaohsiung Chang Gung Memorial Hospital and Chang Gung University College of Medicine, Kaohsiung, Taiwan; ^9^ Division of Hematology-Oncology, Department of Internal Medicine, Kaohsiung Medical University Hospital, Kaohsiung, Taiwan; ^10^ Faculty of Medicine, Kaohsiung Medical University, Kaohsiung, Taiwan

**Keywords:** SLC22A3, cancer therapy, cisplatin, survival, head and neck squamous cell carcinoma

## Abstract

Solute carrier family 22 member 3 (SLC22A3), also called organic cation transporter 3 (OCT3), is responsible for organic cation transport, which can eliminate many endogenous small organic cations, drugs, and toxins. This study investigated whether SLC22A3 expression is related to cisplatin uptake and the survival of patients with head and neck squamous cell carcinoma (HNSCC). Using immunohistochemical staining and digital image analysis, SLC22A3 expression was examined in 42 HNSCC patients who were postoperatively treated with or without adjuvant chemotherapy. SLC22A3-overexpressing SCC-4 cells and SLC22A3-knocked down SCC-25 cells were used to investigate the function of SLC22A3 in cisplatin uptake. We found that patients with higher SLC22A3 expression had longer survival times than those with lower SLC22A3 expression (*p* = 0.051). Moreover, among advanced T-stage patients receiving adjuvant cisplatin therapy, those with higher SLC22A3 expression had longer survival times than those with lower SLC22A3 expression (*p* = 0.006). An *in vitro* study demonstrated that SCC-25 cells with upregulated SLC22A3 expression were more sensitive to cisplatin than were SCC-4 cells with downregulated SLC22A3 expression. An increased uptake of cisplatin and an enhanced cytotoxic effect were observed in SLC22A3-overexpressing SCC-4 cells, and decreased uptake was found in SLC22A3-knocked down SCC-25 cells. Our results demonstrated that upregulated SLC22A3 expression can increase the cisplatin uptake and subsequently improve the survival of patients with HNSCC.

## INTRODUCTION

Head and neck squamous cell carcinoma (HNSCC) is the tenth most common cancer in men worldwide and is currently the seventh most common cause of cancer-related death [1]. The standard treatments for HNSCC consist of surgery, radiotherapy, chemotherapy and combinations of these modalities [2]. Despite aggressive combination treatments, little progress has been made toward improving outcomes [3]. In its early stage, HNSCC may be curable with surgery, radiation, and chemotherapy. However, in the advanced stage, tumor recurrence and metastasis may occur after primary treatment and are associated with a poor outcome.

Solute carrier family 22 member 3 (*SLC22A3*), also called organic cation transporter 3 (*OCT3*), is an imprinted gene located on human chromosome 6. The promoter of *SLC22A3* is located 155 kb from the 3′ end of the *IGF2R* gene, which is closely associated with *SLC22A2/OCT2* and *SLC22A1/OCT1* [4]. SLC transporters (*e.g.* OCT2) are renal membrane transporters regarding its role in cisplatin nephrotoxicity. In mouse models of cisplatin ototoxicity, SLC transporters (*e.g.* CTR1 and OCT2) had been associated with cisplatin-induced ototoxicity. Organic cation/carnitine transporters rOctn1 and rOctn2 may mediate oxaliplatin induced neurotoxicity in rats [5]. SLC transporters expressed in the small intestine, liver, and kidney may play an important role in the disposition of cancer drugs [6] and expressed in cancer cells play an important role in the cellular uptake of anticancer drugs, which may be a determinant step of anticancer drug efficacy [6].

The role of SLC22A3/OCTs in metabolism is the uptake, intracellular inactivation, and biliary or urinary excretion of a broad spectrum of endogenous (e.g., catecholamines) and exogenous substrates (e.g.*,* metformin and betablockers) and anticancer drugs (e.g., platin derivatives) [7–12]. Therefore, *SLC22A3/OCTs* may be associated with HNSCC and its prognosis.

Platinum agents are widely used in the treatment of cancer. Cisplatin (cis-diamminedichloroplatinum II, CDDP) was the first platinum agent to be synthesized, and it has since played an essential role in cancer chemotherapy for 30 years. However, cisplatin efficacy is far from optimal because of its poor tumor cell specificity and its hydrophilic properties, which limit the intracellular uptake of cisplatin. Therefore, developing strategies to improve its intracellular uptake may improve cisplatin efficacy and patient survival.

We hypothesized that the expression level of *SLC22A3* influences the anticancer effect of cisplatin against HNSCC. Therefore, in this study, we elucidated the effect of *SLC22A3* expression on cisplatin uptake by HNSCC cells with overexpression or knockdown of *SLC22A3*. The survival was assessed to evaluate the response to chemotherapy of patients and to determine the correlation between the response and their expression levels of *SLC22A3*.

## RESULTS

### Patients with higher SLC22A3 expression had an improved survival rate after cisplatin therapy

**Figure 1:**
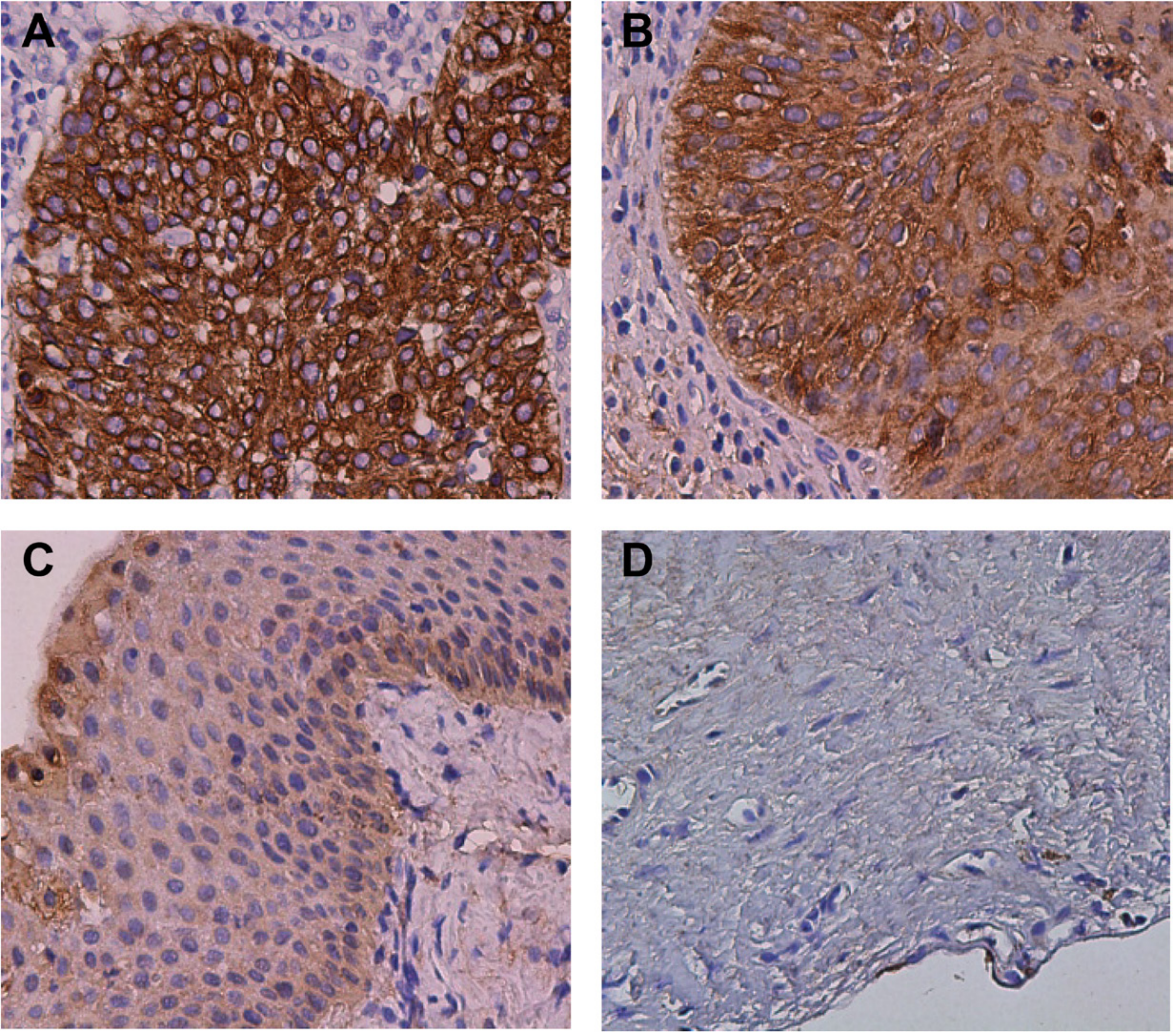
Immunohistochemical staining of SLC22A3 in HNSCC Representative images of HNSCC show (**A**) high positive SLC22A3 expression (score = +3) and (**B**) positive SLC22A3 expression (score = +2) in cancerous tissues and (**C**) low positive SLC22A3 expression (score = +1) and (**D**) negative SLC22A3 expression (score = 0) in adjacent noncancerous tissues. Original magnification: ×200.

**Figure 2:**
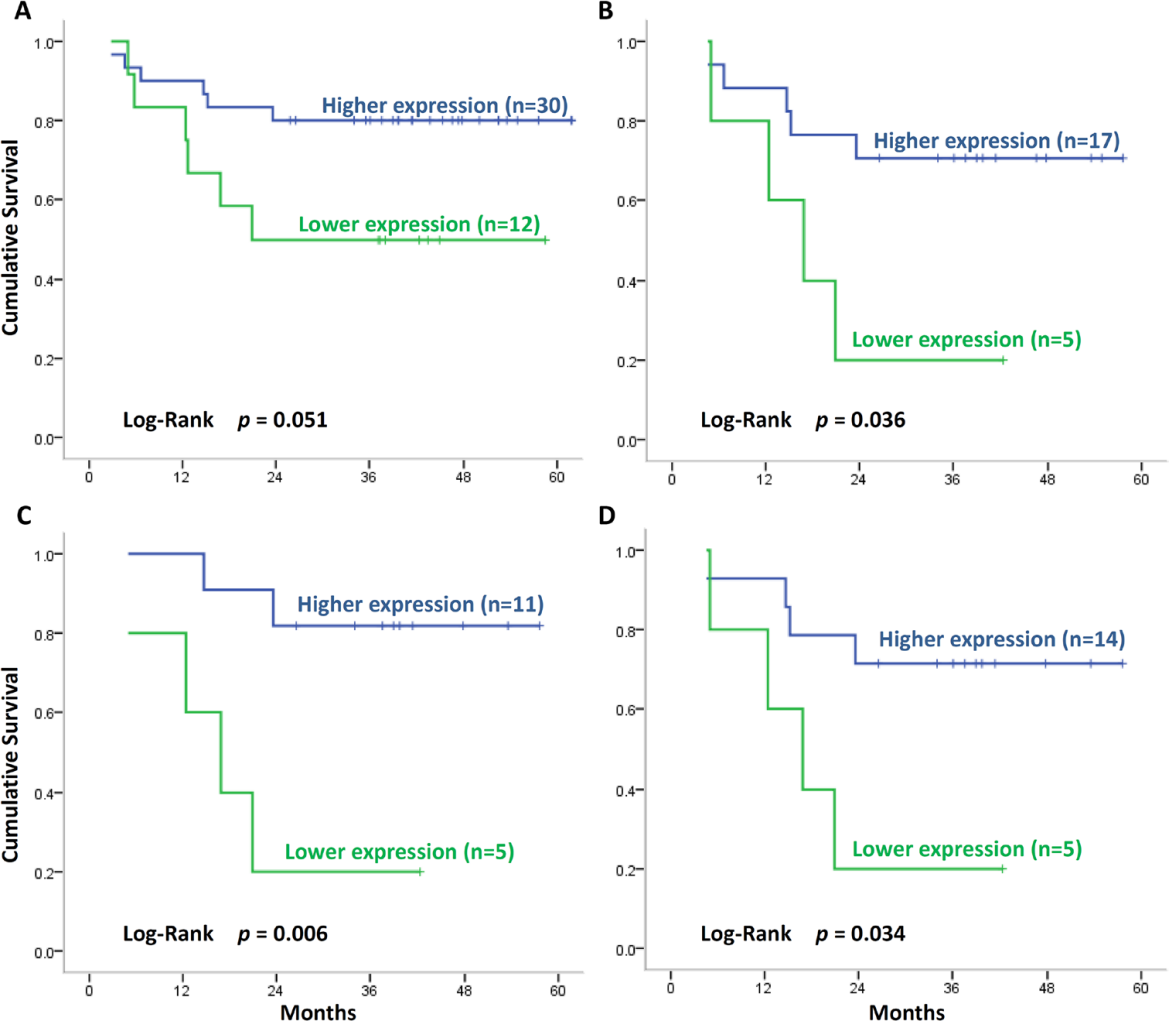
Survival of patients with HNSCC and SLC22A3 expression (**A**) Survival of patients with higher SLC22A3 expression (score difference = 1 or 2) and lower SLC22A3 expression (score difference = 0). Patients with higher SLC22A3 expression exhibited improved survival. (**B**) Survival of patients receiving cisplatin therapy. Patients with higher SLC22A3 expression exhibited improved survival after cisplatin therapy. (**C**) Survival of advanced *T*-stage patients receiving cisplatin therapy. Advanced T-stage patients with higher SLC22A3 expression exhibited improved survival after cisplatin therapy. (**D**) Survival of advanced clinical stage patients receiving cisplatin therapy. Advanced clinical stage patients with higher SLC22A3 expression exhibited improved survival after cisplatin therapy.

In this study, we investigated SLC22A3 protein expression in patients with HNSCC by using IHC staining. Table 1 lists the clinical pathological characteristics of the 42 patients enrolled in this study. Figure 1 shows IHC staining for different expression levels of SLC22A3 and the corresponding scores. Furthermore, patients were divided into two groups according to the difference in the scores of tumorous and adjacent normal tissue. The higher SLC22A3 expression group was defined as a score difference of 1 or 2, which implies higher expression in tumorous tissue than in adjacent normal tissue. The lower SLC22A3 expression group was defined as a score difference of 0, which implies the same level of SLC22A3 expression in tumorous and adjacent normal tissues. A total of 30 and 12 patients were included in the higher and lower expression group, respectively (Table 2). The 2-year survival rate in the higher expression group was higher than that in the lower expression group (80.0% vs. 50.0%, *p* = 0.051) (Figure 2A). According to the head and neck cancer guideline of our hospital, 22 high-risk patients received adjuvant therapies including 5-fluorouracil (FU) combined with cisplatin (*n* = 15) or cisplatin only (*n* = 7). We found that among patients receiving cisplatin therapy, those with higher SLC22A3 expression had a higher 2-year survival rate than those with lower SLC22A3 expression (*p* = 0.036) (Figure 2B). Further analysis revealed that advanced T-stage patients (Figure 2C) and advanced clinical stage patients (Figure 2D) with higher SLC22A3 expression also had improved survival after cisplatin therapy (*p* = 0.006 and *p* = 0.034, respectively).

**Table 1:** Characteristics of patients with HNSCC

Characteristic	No. of patients
Sex Male Female Median age, y (range)	40 2 53.7 (30–76)
T stage T1 T2 T3 T4a T4b	5 13 6 16 1
N stage N0 N1 N2a N2b N2c	25 3 2 6 6
Primary tumor site Oral cavity Larynx Supraglottic Ororpharynx Hypopharynx	26 1 2 9 6
Tumor size < 3 cm > 3 cm	20 22
Neck metastasis Positive Negative	17 25
Two-year survival Expired Survived	12 30

**Table 2:** Characteristics of patients with HNSCC stratified by SLC22A3 expression level

**Characteristic**	**SLC22A3** **Higher expression**	**SLC22A3** **Lower expression**	***p* value**
No. of patients	30 (71.4%)	12 (28.6%)	
Gender Male/Female	28/2	12/0	0.999
Median age, y (range)	55.3 (38–76)	50.0 (30–70)	0.135
T Stage T1 + T2 T3 + T4	14 16	5 7	0.999
Tumor size > 3 cm	11 (36.7%)	6 (50.0%)	0.426
Two-year survival Survival/Expired	24/6 (80.0%)	6/6 (50.0%)	0.052
Treatment Cisplatin Non-cisplatin	17 (56.7%) 13 (43.3%)	5 (41.7%) 7 (58.3%)	0.499

a) Higher expression: score difference = 1 or 2

b) Lower expression: score difference = 0

c) Data are expressed as the mean ± SD.

### Sensitivity to cisplatin was higher in SLC22A3 higher expressing SCC-25 cells than in SLC22A3 lower expressing SCC-4 cells

**Figure 3:**
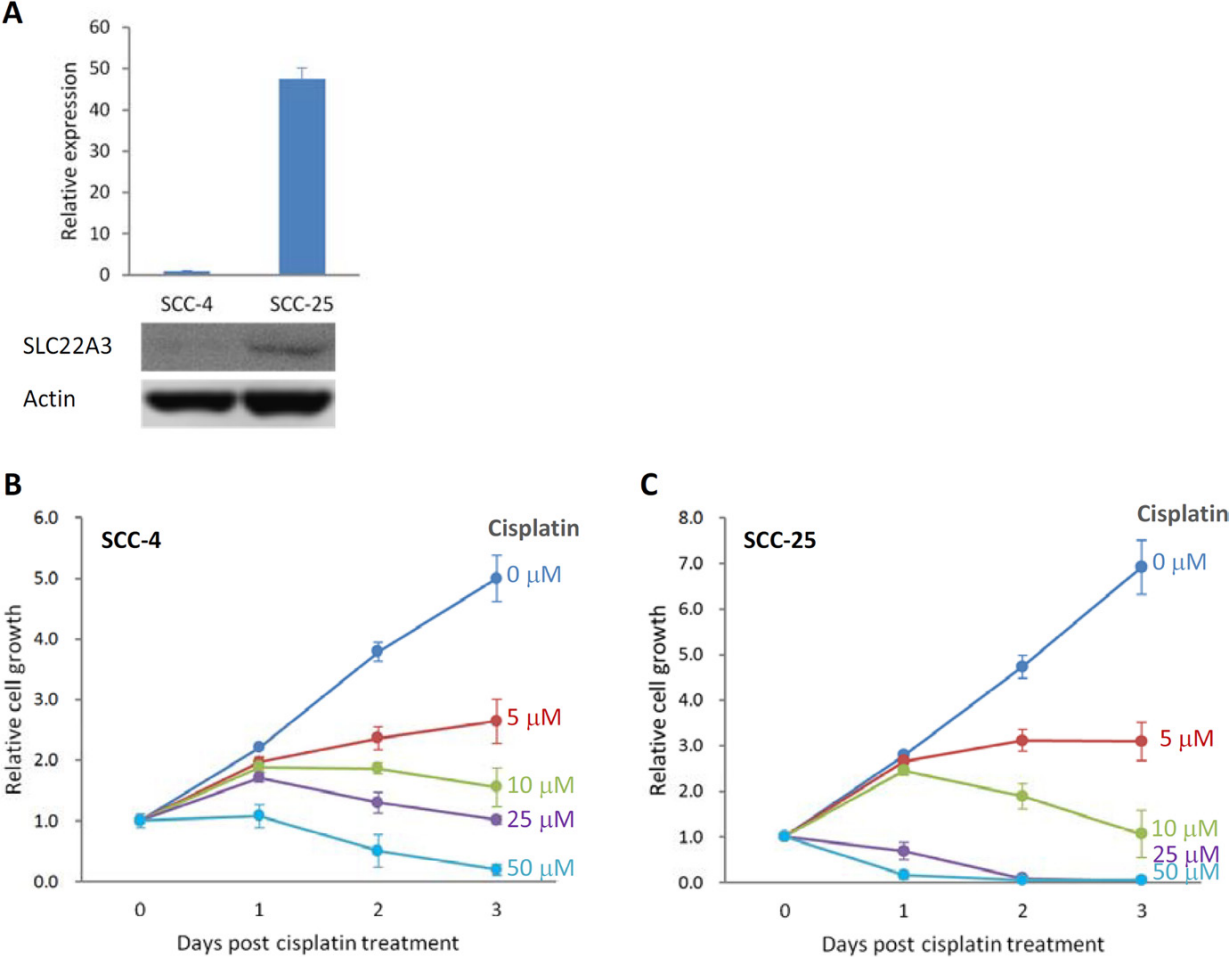
Sensitivity to cisplatin correlated with the level of SLC22A3 expression (**A**) SLC22A3 expression was lower in SCC-4 cells but higher in SCC-25 cells, as determined by qRT-PCR (upper panel) and Western blotting (lower panel). The value of *SLC22A3* expression in SCC-4 cells is designated 1, and the expression level in SCC-25 cells is related to this value. An MTT assay was performed to examine the effect of cell toxicity for SCC-4 cells (**B**) and SCC-25 cells (**C**) after treatment with various concentrations of cisplatin for 3 days. The relative cell growth is presented by comparing the data with day 0. Data are presented as the mean and SE of three independent experiments.

We examined the expression level-dependent effect of *SLC22A3* on cisplatin-induced cytotoxicity. Typically, SCC-25 cells exhibit higher *SLC22A3* expression than SCC-4 cells (Figure 3A). Through an MTT assay, we found that the IC_50_ of cisplatin for SCC-4 cells was approximately 25 μM at 3 days after treatment (Figure. 3B). However, after SCC-25 cells were treated with 10 μM cisplatin, cell growth decreased drastically at 3 days after treatment (Figure 3C).

### Cytotoxic effect of cisplatin was enhanced by SLC22A3 overexpression in SCC-4 cells

**Figure 4:**
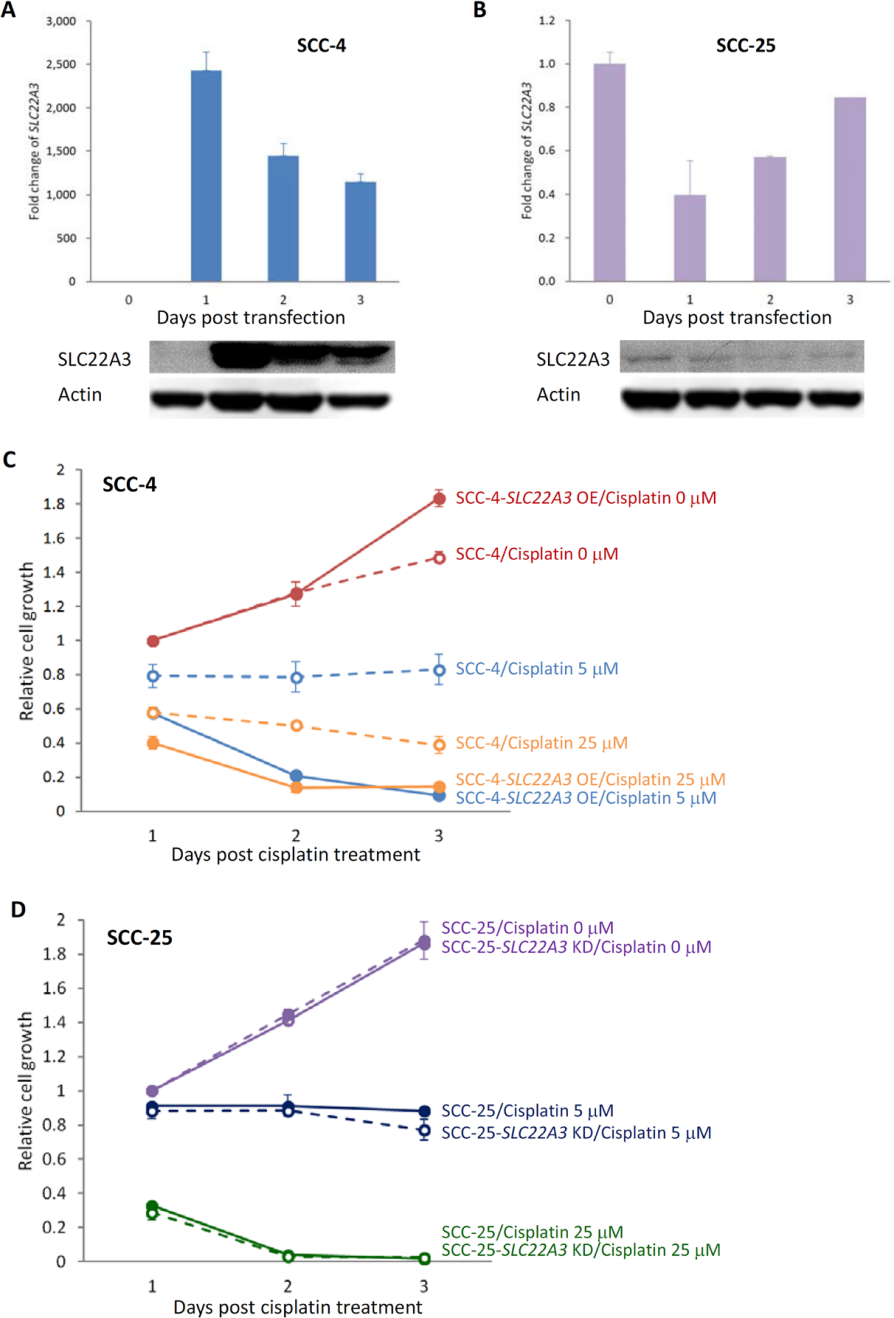
Cytotoxic effect of cisplatin was enhanced by SLC22A3 overexpression (**A**) *SLC22A3* expression was upregulated in SCC-4 cells transfected with pCMV6-AC-MycDDK plasmid vector containing *SLC22A3,* as determined by qRT-PCR (upper panel) and Western blotting (lower panel). (**B**) *SLC22A3* expression was downregulated in SCC-25 cells transfected with *SLC22A3* siRNA, as determined by qRT-PCR (upper panel) and Western blotting (lower panel). The value of *SLC22A3* expression on the day of transfection is designated 1, and the expression levels on different days after transfection are related to this value. An MTT assay was performed to examine the the effect of cell toxicity for SCC-4 cells (**C**) and SCC-25 cells (**D**) at 3 days after transfection and treatment with 0, 5, and 25 mM cisplatin. Data are presented as the mean and SE of three independent experiments.

To elucidate the effects of *SLC22A3* expression on cisplatin-induced cytotoxicity, we transiently overexpressed *SLC22A3* in SCC-4 cells (Figure 4A) and knocked down *SLC22A3* in SCC-25 cells (Figure 4B). When *SLC22A3*-overexpressing SCC-4 cells were treated with 5 and 25 μM cisplatin for 72 hours, cisplatin-induced cytotoxicity was strongly enhanced by *SLC22A3* expression (Figure 4C). However, cisplatin-induced cytotoxicity did not differ between SCC-25 cells with or without *SLC22A3* knockdown (Figure 4D).

### Transport of cisplatin

**Figure 5:**
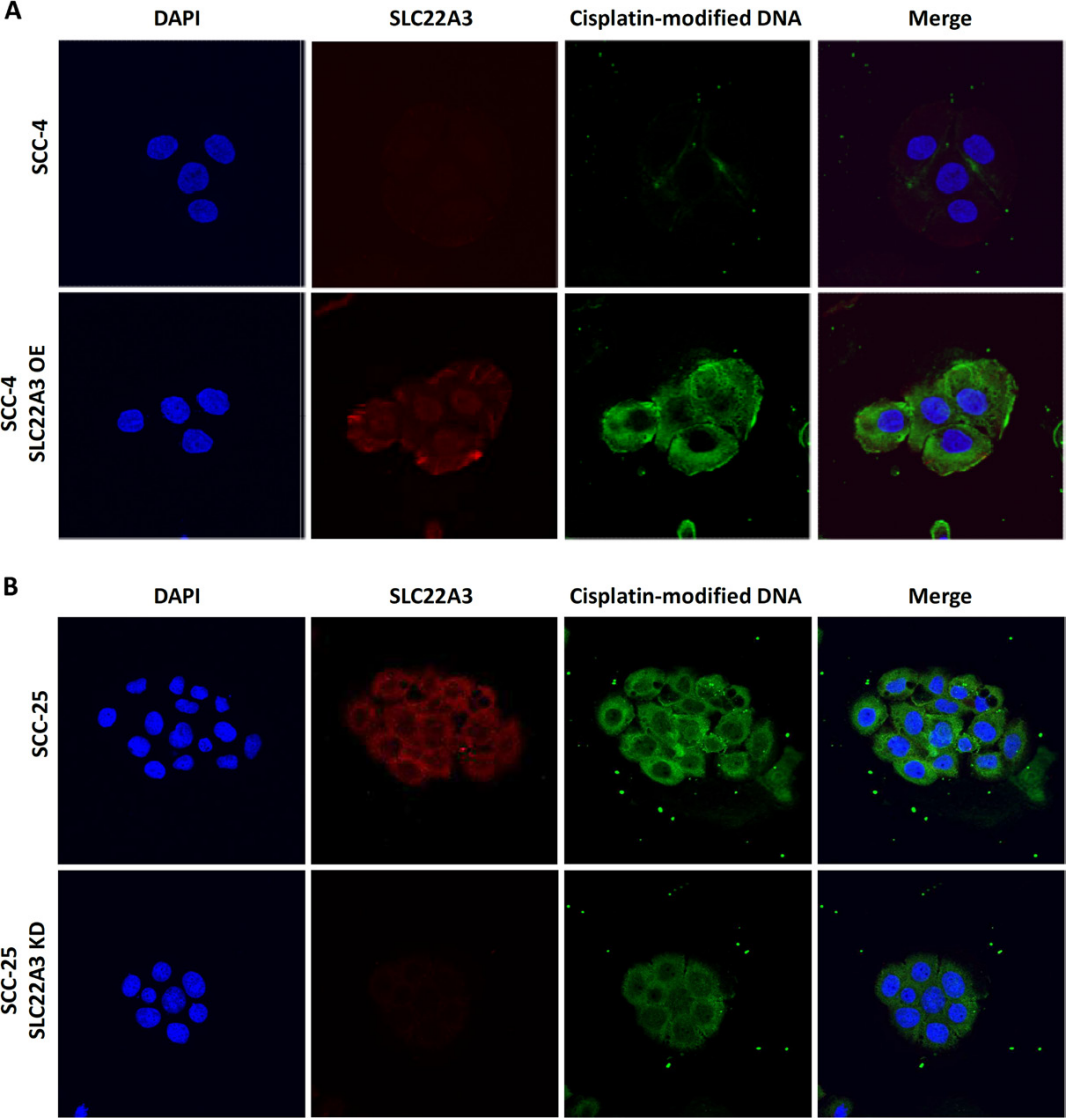
Uptake of cisplatin by SCC-4 and SCC-25 cells (**A**) SCC-4 and *SLC22A3 (OCT)*-overexpressing SCC-4 cells and (**B**) SCC-25 and *SLC22A3 (OCT)*-knocked down SCC-25 cells were treated with 50 μM cisplatin for 6 hours. Confocal microscopy was performed using anti-cisplatin-modified DNA antibody (primary antibody) plus TRITC-labelled goat anti-rabbit IgG (secondary antibody) and anti-SLC22A3 (OCT) antibody (primary antibody) plus Alexa Fluor 488-labelled goat anti-rat IgG (secondary antibody). DNA was counterstained with DAPI. The red signal represents SLC22A3 protein and the blue signal represents nucleus. The green signal represents influx of cisplatin-binding DNA.

To further clarify the effect of SLC22A3 on the uptake of cisplatin, we examined the uptake of cisplatin in *SLC22A3*-overexpressing SCC-4 cells and *SLC22A3*-knocked down SCC-25 cells. We observed increased uptake of cisplatin in *SLC22A3-*overexpressing SCC-4 cells (Figure 5A). However, decreased uptake of cisplatin was observed in *SLC22A3-*knocked down SCC-25 cells (Figure 5B).

## DISCUSSION

*SLC22A3* mRNA has been detected in the placenta, intestine, heart, brain, and kidney, but the distribution of *SLC22A3* in the plasma membrane and its physiological role are not yet clearly understood [13–15]. A recent study demonstrated that OCT1 and OCT3 play a role in the uptake of serotonin and histamine and metformin in the gastrointestinal tract [16].

*SLC22A3* has been extensively studied in many tissues and tumor cell lines and has been found to be associated with breast cancer, bladder cancer, and lung cancer [17–20]. However, only a few studies have investigated *SLC22A3* expression and its role in human malignancies including HNSCC.

In the present study, we graded the expression level of SLC22A3 according to IHC staining by using digital image analysis, and patients were divided into a higher SLC22A3 expression group (higher SLC22A3 expression in tumorous tissue than in normal tissue) and a lower SLC22A3 expression group (the same level of SLC22A3 expression in tumorous and normal tissues). We found a significant correlation between SLC22A3 expression and the response to adjuvant chemotherapy. Therefore, we hypothesized that the substrate specificity and expression level of SLC22A3 affect the anticancer effect of platinum agents against HNSCC. OCTs may play an important role in the treatment of malignant tumors because they are responsible for the cytotoxicity of platinum derivatives and are predictors of responses to small molecules [21]. Cisplatin plays an essential role in chemotherapy against solid tumors of the prostate, bladder, lung, and testis and HNSCC [22]. However, its efficacy is limited by its hydrophilic properties, which limit its intracellular uptake [23].

Cisplatin is activated when it enters the cell. It binds to the N7 reactive center on purine residues and can cause DNA damage in cancer cells, inhibiting cell division and resulting in apoptotic cell death [22]. The cisplatin–DNA adduct level is the most critical determinant of the sensitivity of HNSCC cells to cisplatin [24].

Cisplatin is an important chemotherapeutic against solid tumors of the prostate, bladder, lung, testis, liver, and brain [25]. Concurrent treatment with high-dose cisplatin and radiotherapy represents the definitive adjuvant treatment for high-risk HNSCC [26]. However, the effect of cisplatin on recurrent HNSCC is weak. Passive transporters have been implicated to play a role in the cellular influx of cisplatin and carboplatin. The *SLC* gene encodes a large family of passive transporters predominated by ion-coupled transporters and exchangers. These transporters include OCTs, which are highly expressed in the proximal tubules of the kidney and appear to be integral to development [15]. Recent studies have identified OCT as a membrane transporter capable of transporting cisplatin into cells. Therefore, a high level of SLC22A3 may benefit generating a favorable response to platin treatment. Similarly, a recent study showed that SLC22A3 expression in renal cell carcinoma cell lines also enhanced sensitivity to chemotherapeutics such as melphalan, irinotecan, and vincristine [27]. This finding may explain why the survival of patients in the higher expression group had improved after cisplatin treatment.

In this study, we found that the expression of *SLC22A3* was low in SCC-4 cells and was high in SCC-25 cells. Sensitivity to cisplatin also differed between the two cell lines. SCC-25 cells with higher levels of *SLC22A3* expression showed high sensitivity and strong cisplatin-induced cytotoxicity, whereas SCC-4 cells with lower *SLC22A3* expression showed low sensitivity and weak cisplatin-induced cytotoxicity.

In a functional study, we found that upregulated *SLC22A3* expression in SCC-4 cells increased sensitivity to cisplatin and enhanced cisplatin-induced cytotoxicity. However, the knockdown of *SLC22A3* in SCC-25 cells did not reduce sensitivity to cisplatin or cisplatin-induced cytotoxicity. This finding may be because SLC22A3 is not the only cisplatin transporter or channel. A recent study demonstrated that copper transporter 1 (CTR1) markedly influenced the uptake of all clinically used platinum-containing drugs, suggesting that CTR1 also transports DDP [28, 29]. Therefore, despite *SLC22A3* knockdown, cisplatin may still enter the cells through CTR1.

In the uptake study, we found that SCC-4 cells with upregulated *SLC22A3* expression exhibited increased uptake of cisplatin, which may explain the accompanying increased sensitivity to cisplatin and enhanced cisplatin-induced cytotoxicity. Hence, the efficacy of cisplatin treatment may be higher in patients with higher *SLC22A3* expression, and these patients may subsequently exhibit improved survival. By contrast, SCC-25 cells with the knockdown of *SLC22A3* exhibited a reduced uptake of cisplatin. However, the reduced uptake of cisplatin by closing the SLC22A3 channel was insufficient to fully enhance resistance to cisplatin because the uptake of cisplatin may occur through the alternative transporter CTR1.

We speculate that when the expression of SLC22A3 is increased in recurrent or metastatic tumors, these tumors may become platinum-resistant. In our study, we enrolled only primary tumor and excluded the recurrent and metastatic tumors, therefore we have no direct evidence to know the changes of SLC22A3 expression occurring in recurrent tumors or metastatic tumors *vs.* primary tumors. It has been suggested that the development of acquired platinum resistance involved epithelial to mesenchymal transition (EMT), resulting in tumor aggressiveness with motile function of cancer cells [30]. Whether SLC22A3 is also involved in the development of acquired platinum resistance will be the main issue for our next study to explore and we can start from comparing the differential SLC22A3 expression between recurrent tumor and primary tumor.

We hypothesized that higher *SLC22A3* expression in cancer cells may enhance the uptake of cisplatin after cisplatin treatment and, in turn, reduce the survival of cancer cells, and the opposite may hold true for cancer cells with lower *SLC22A3* expression*.* Therefore, *SLC22A3* may be a novel therapeutic marker for patients with HNSCC receiving cisplatin-based chemotherapy. IHC staining of tumorous tissue samples by using SLC22A3 antibody and the quantification of the immunostained samples are also required to establish a parameter for clinical decision-making processes, particularly for patients requiring adjuvant treatment.

Our future goal is to establish screening methods for SLC22A3 expression in peripheral blood samples or biopsied tissues to predict the response to and outcome of cisplatin treatment. Imaging with radiolabeled cisplatin might have major associations with the treatment outcome. If this can be accomplished, we can identify the poor responders to cisplatin treatment and then select a more efficient therapy, such as targeted therapy or immunotherapy, for these individuals. We also hope to identify the activators of SLC22A3 expression to enhance the uptake of cisplatin by cancer cells. However, as the effect of anticancer effects are enhanced by the up-regulated SLC22A3, the side effects of chemotherapy may also be enhanced. So we need to be more careful in the future application.

In conclusion, the study findings suggest that cisplatin-induced cytotoxicity is mediated by the uptake of cisplatin by cancer cells through SLC22A3, and SLC22A3 expression in cancer cells is a potential biomarker for including cisplatin in cancer chemotherapy. Our findings reveal some strategies for improving the treatment outcome for advanced HNSCC.

## MATERIALS AND METHODS

### Patients and samples

Cancerous tissues and adjacent noncancerous tissues were obtained from 42 patients diagnosed as having HNSCC who were undergoing surgery at the Department of Otolaryngology, Kaohsiung Chang Gung Memorial Hospital, Taiwan, between 2011 and 2013. Immediately after resection, the obtained specimens were snap-frozen in liquid nitrogen and stored at −80°C until use. Prior to tissue acquisition, informed consent was obtained from all enrolled patients. This study was approved by the Institutional Review Board of Chang Gung Memorial Hospital (IRB Approval No. 100-4455A3).

### Disease severity and treatment of patients with HNSCC

The TNM staging system was established by the American Joint Committee on Cancer and includes the tumor (T), neck lymph node (N), and metastasis (M) status. Stages I–IV represent the general cancer status from mild (stage I) to severe (stage IV) and are closely related to prognosis and the therapy response. In the present study, we divided the enrolled patients into two disease severity groups for a correlation analysis of *SLC22A3* expression: (1) early stage (stages I and II) and advanced stage (stages III and IV) groups, and (2) tumor stage T1–T2 and T3–T4 groups. Patients with HNSCC were treated in accordance with the head and neck cancer guidelines of the National Comprehensive Cancer Network: cisplatin at dosages of 60–75 mg/m^2^ by intravenous infusion for 4 hours with or without 5-FU at dosages of 600–750 mg/m^2^ per 24 hours as a 96-hour continuous intravenous infusion, repeated every 3 weeks per cycle. The survival status of patients was followed up for at least 2 years after surgery. Overall survival was calculated from the date of diagnosis until death or last follow-up.

### Immunohistochemical (IHC) staining of SLC22A3

IHC staining of cancerous tissues and adjacent noncancerous tissues from patients with HNSCC was performed. Tissue sections were incubated with monoclonal antibody against SLC22A3 (1:200 dilution; Epitomics, Burlingame, CA, USA) for 1 hour and then incubated with biotinylated goat anti-rabbit antibody for 30 minutes. Finally, the specific binding was visualized using a horseradish peroxidase-diaminobenzidine staining kit (Abcam Inc., MA, USA).

### Digital image analysis

All IHC-stained slides were observed under a light microscope (Zeiss, Gottingen, Germany) at ×400 magnification. Four 2-dimensional images were taken for each slide by using a microscope camera (Zeiss). To assess SLC22A3 expression, the percentage of area and integrated density (staining intensity) were calculated using ImageJ free software, version 1.410 (NIH, Maryland, USA). The relative intensity of IHC staining was quantified using ImageJ64 and was normalized to that of an internal control. According to the standard grading procedure, staining intensity was assigned scores using a 4-tier scoring system; that is, 3+, high positive; 2+, positive; 1+, low positive; and 0, negative. Subsequently, patients were divided into three groups according to the difference in the scores assigned to tumorous and normal tissues; that is, score differences of 2, 1, and 0, in a blinded manner.

### Cell cultures

Two human HNSCC cell lines, SCC-4 and SCC-25 (both tongue squamous cell carcinoma cell lines), used in this study were purchased from Food Industry Research and Development Institute, Taiwan. These cells were maintained in MEM (Minimum Essential Medium Eagle)-F12 medium (Invitrogen, Carlsbad, CA, USA) containing 0.4 μg/mL hydrocortisone (Sigma-Aldrich, St. Louis, MO, USA) and 10% FBS and were grown at 37°C with 5% CO_2_.

### MTT assay

SCC-4 and SCC-25 cells were treated with various concentrations of cisplatin or PBS (Phosphate-Buffered Saline, as control); subsequently, the percentages of metabolically active cells were determined on the basis of the mitochondrial conversion of MTT into formazine. In brief, after cells were treated with or without cisplatin for different incubation times, culture media were replaced with DMEM/F-12 (Dulbecco’s Modified Eagle’s Medium, without phenol) containing 0.02% MTT (Sigma-Aldrich) and incubated for 4 hours; subsequently, the medium was replaced with 200 μL of dimethyl sulfoxide per well. The results were assessed in a 96-well format plate reader by measuring the absorbance at a wavelength of 595 nm on a DTX880 Multimode Detector (Beckman Coulter, Brea, CA, USA).

### Transfection

For the transient expression and knockdown of *SLC22A3*, pCMV6-AC-MycDDK plasmid vector DNA containing *SLC22A3* (OriGene Technologies, Rockville, MD, USA) and *SLC22A3* Stealth siRNA (ThermoFisher Scientific, NY, USA) were used, respectively. Cells (4000 cells/well) were transfected with 3 µg of *SLC22A3* overexpression plasmid DNA or *SLC22A3* siRNA per well by using 1 μL of Lipofectamine 2000 (Invitrogen), according to the manufacturer’s instructions. After transfection for 30 minutes, cells were treated with various concentrations of cisplatin (0, 5, 10, 25, and 50 μM) and were harvested on days 1, 2, and 3 after transfection.

### Cisplatin uptake and immunofluorescence staining

SCC-4 and SCC-25 cells were transfected with *SLC22A3*-overexpressing plasmids and *SLC22A3* siRNA, respectively. On the next day, cells were cultured for 6 hours in a medium containing 50 μM cisplatin. Cells were then fixed with 4% paraformaldehyde, penetrated by freezing at −80°C, and permeated with 0.5% Triton X-100 in PBS. After cells were blocked with 1% bovine serum albumin, they were stained with rat anti-cisplatin modified DNA antibody [CP9/19] (1:100 dilution; Abcam) for 16 hours at room temperature and were then stained with rabbit anti-SLC22A3 antibody (1:100 dilution; Abcam) for 4 hours. The secondary antibodies of goat anti-rabbit IgG labeled with TRITC (Sigma-Aldrich) and goat anti-rat IgG labeled with Alexa Fluor 488 (Abcam) were added at 1:400 dilution and incubated at room temperature for 1 hour. Slides were mounted with a mounting solution containing DAPI (4′,6-Diamidino-2-Phenylindole, Dilactate) (Vector Lab, Burlingame, CA, USA) and were then analyzed using a confocal microscope (Fluoview FV10i; Olympus, Tokyo, Japan).

### Statistical analyses

Kaplan–Meier survival curves were compared using a log-rank test. The Pearson chi-square test was used to determine the differences among categorical variables, and the Student *t* test and ANOVA were used to determine the differences among continuous variables. All tests were two-sided, and results with *p* < 0.05 were considered statistically significant. All statistical analyses were performed using SPSS version 13.0 for Windows (SPSS, Chicago, IL, USA).
